# IGFBP3, a Transcriptional Target of Homeobox D10, Is Correlated with the Prognosis of Gastric Cancer

**DOI:** 10.1371/journal.pone.0081423

**Published:** 2013-12-27

**Authors:** Meng Xue, Yanfei Fang, Guoming Sun, Wei Zhuo, Jing Zhong, Cuijuan Qian, Lan Wang, Liangjing Wang, Jianmin Si, Shujie Chen

**Affiliations:** 1 Department of Gastroenterology, Sir Runrun Shaw Hospital, Zhejiang University, Hangzhou, Zhejiang, China; 2 Institute of Gastroenterology, Zhejiang University, Hangzhou, Zhejiang, China; 3 Department of Gastroenterology, Second Affiliated Hospital, Zhejiang University, Hangzhou, Zhejiang, China; Vanderbilt University Medical Center, United States of America

## Abstract

Homeobox D10 (HoxD10) plays important roles in the differentiation of embryonic cells and progression of breast cancer. Our previous report revealed that insulin-like growth factor binding protein-3 (IGFBP3) was regulated by HoxD10 in gastric cancer cells; however, the functional roles and underlying mechanisms of IGFBP3 in gastric cancer remain unclear. Here, we found that the expression of IGFBP3 were upregulated after ectopic expression of HoxD10 in gastric cancer cells. Chromatin immunoprecipitation assay showed that HoxD10 bound to three potential regions of IGFBP3 promoter. Exogenous HoxD10 significantly enhanced the activity of luciferase reporter containing these binding regions in gastric cancer cells. Further data showed that all of these binding sites had Hox binding element “TTAT”. Immunohistochemical staining results revealed that IGFBP3 expression was significantly downregulated in 86 gastric adenocarcinomas tissues relative to their adjacent non-cancerous tissues (p<0.001). Moreover, IGFBP3 expression was significantly lower in gastric tumor with lymph node metastasis compared with that without lymph node metastasis (p=0.045). Patients with high expression level of IGFBP3 showed favorable 5 year overall survival (p=0.011). Knockdown of IGFBP3 accelerated gastric cancer cell migration and invasion and induced the expression of invasive factors including MMP14, uPA and uPAR. Thus, our data suggest that HoxD10-targeted gene IGFBP3 may suppress gastric cancer cell invasion and favors the survival of gastric cancer patients.

## Introduction

Gastric cancer is the fourth most frequent cancer and currently is the second leading cause of cancer-related death worldwide [1]. Nearly half of the gastric caner patients have already advanced to the late stage with lymphatic or distant metastasis at diagnosis and lose the opportunity for surgery [2]. These patients have a poor outcome and the five-year survival rate for those with distant metastasis is less than 5% [3]. The progression of gastric cancer is considered to be a multi-step process that involves the activation of oncogenes and inactivation of tumor suppressor genes. We have previously presented that homeobox D10 (HoxD10) served as a tumor suppressor by suppressing tumor growth and invasiveness, which was epigenetically silenced in gastric cancer [4]. 

HoxD10 gene belongs to the homeobox superfamily, which encode transcription factors and exert functions mainly through the activation or repression of downstream target genes [5]. Amino-terminal arm of the Hox homeodomain has several core consensus binding elements, including TTAT, TAAT and TTAC [6,7]. For instance, HoxC8 binds to these elements at promoter regions and modulates the transcriptional activities of pedf, Ncam, Zac1, Opn and Cdh11, as determined by high-throughout chromatin immunoprecipitation assays and global HoxC8 DNA-binding site analysis [7]. Studies have demonstrated that HoxD10 inhibits the angiogenesis and cell motility in endometrial cancer (EC) [8] and impairs the cell invasiveness of gastric and breast cancers [4,9]. However, little is known about the mechanisms of how HoxD10 exerts its functions in carcinogenesis and tumor progression. We have systematically screened the potential targets of HoxD10 by cDNA microarray and identified multiple genes, including insulin-like growth factor binding protein-3 (IGFBP3) that might be responsible for the tumor suppressing effect of HoxD10 in gastric cancer [4]. 

IGFBP3 is a major IGFBP species in circulation, binding over 75% of circulating insulin growth factor-I (IGF-I) [10]. It could inhibit cell proliferation and induce cell apoptosis of several types of cancer, including prostate and gastric cancers [11,12]. Several studies have confirmed that IGFBP3 suppresses the invasiveness of endometrial cancer (EC) cells [13], metastasis in prostate cancer [14], and angiogenesis in head and neck squamous cell carcinoma (HNSCC) [15]. It was generally considered that IGFBP3 could block the binding region of IGF-I on its receptor IGF-IR, thus abolish the effect of IGF/IGF-IR axis. Besides the dependence of IGF/IGF-IR axis, there exists alternative IGF/IGF-IR independent and nuclear translocation ways [10]. Many factors have been clarified to regulate the expression of IGFBP3, including retinoic acid, tumor necrosis factor-α, trichostatin A, caudal type homeobox 2 (CDX2) and the methylation status of itself [10,16]. Our previous studies showed that IGFBP3 was upregulated in AGS and MKN28 cells overexpressing HoxD10 [4]. Considering promoter region of IGFBP3 has several potential binding sites for HoxD10, predicted by PROMO, a program for the prediction of transcription factor binding sites [17], HoxD10 might directly interplay with IGFBP3 in the regulation of gastric cancer cell invasion. Elucidation of this network would provide further insights into the role of IGFBP3 in gastric cancer. 

In the present study, we identified that HoxD10 could directly bind the promoter regions of IGFBP3 potentially via the TTAT element, thus transcriptionally regulates its expression in gastric cancer. In addition, the expression level of IGFBP3 is frequently downregulated in gastric cancer tissues and related to the overall survival, suggesting that IGFBP3 plays an important role in gastric cancer progression. Functionally, IGFBP3 could suppress the migration and invasion of gastric cancer cells, at least in part, through the regulation of those invasive factors, including metalloproteinase-14 (MMP14) and urokinase-type plasminogen activator (uPA).

## Materials and Methods

### Cell culture

Eight gastric cancer cell lines (AGS, MKN28, MKN45, NCI-N87, BGC823, HGC27, MGC803 and SGC7901) were obtained from Riken Gene Bank (Tsukuba, Japan) and American Type Culture Collection (Manassas, USA). One non-malignant gastric cell line (GES-1) was obtained from Beijing Institute for Cancer Research (Beijing, China). Cells were cultured in RPMI 1640 medium (Invitrogen, Carlsbad, USA) supplemented with 10% fetal bovine serum (FBS, Sijiqing, Huzhou, China), and incubated at 5% CO_2_, 37°C and 95% humidity.

### Construction of IGFBP3 luciferase reporter plasmids

Three different fragments in the promoter region of IGFBP3, including -2282 to +56 bp (2.3 kb, pIGFBP3), -2251 ~ -2073 bp (HoxD10 binding site I, HBSI) and -1727 ~ -943 bp (HBSII), were amplified by PCR using genomic DNA extracted from HEK-293T wild cells as the template. HBSI encompasses HBS1 and 2, HBSII contains of HBS3, 4 and 5, in which HBS1-5 are predicted by PROMO (http://alggen.lsi.upc.es/) [17]. HBS primer sequences were as follows: pIGFBP3, 5’-GGGGTACCCTGGACGCCTGGAGCTTT-3’ and 5’-GAAGATCTAGCAGCACCAGCAGAGTC-3’; HBSI, 5’-GGGGTACCCATTCGGCACTGAACAAG-3’ and 5’-GAAGATCTAGCCTGGACTGACCACTG-3’; HBSII, 5’-GGGGTACCGGCACTCCATTGTTCTT-3’ and 5’-GAAGATCTGAATAATAAAGACAATAAACTGG-3’. The PCR fragments were ligated into pGM-T vector (Tiangen, Beijing, China), digested with KpnI/BgIII (Promega, Madison, USA) and then subcloned into the KpnI/BgIII sites in the pGL3-basic vector (Promeg) to generate pGL3-pIGFBP3-basic luciferase reporter plasmids, and the pGL3-promoter vector (Promega) to generate pGL3-HBSI (II)-promoter luciferase reporter plasmids. All constructs were confirmed by DNA sequencing.

### Construction of luciferase reporter plasmids of HBSs

We synthesized oligonucleotides with predicted Hox binding sites (HBSs) in the promoter regions of IGFBP3. HBS3 (-1700 ~ -1691bp) , HBS4 (-1418 ~ -1409bp), HBS5 (-953bp ~ -944bp) and each with single point mutant site (A → C), oligonucleotides were as follows: 

HBS3, 5’-CTCTTTTTATTA-3’ and 5’-CATGGAGAAAAATAATCTAG-3’; 

mutant HBS3, 5’-CTCTTTTT**C**TTA-3’ and 5’-CATGGAGAAAAA**G**AATCTAG-3’; 

HBS4, 5’-CATTTGCTATTA-3’ and 5’-CATGGTAAACGATAATCTAG-3’; 

mutant HBS4, 5’-CCTTTGCT**C**TTA-3’ and 5’-CATGGGAAACGA**G**AATCTAG-3’; 

HBS5, 5’-CCTTTATTATTA-3’ and 5’-CATGGGAAATAATAATCTAG-3’; 

mutant HBS5, 5’-CCTTTCTT**C**TTA-3’ and 5’-CATGGGAAAGAA**G**AATCTAG-3’. 

The double-stranded HBSs were annealed and inserted into the upstream of pGL3-promoter vector to generate luciferase reporter plasmids [18]. All constructs were confirmed by DNA sequencing.

### Cell transfection

Gastric cacner cells (BGC823 and SGC7901) were transfected with pcDNA3.1-HoxD10 or pcDNA3.1 empty vector using Fugene HD (Promega). To generate stable HoxD10 overexpression cell lines, above transfected cells were selected by 400 μg/ml G418 (Merck, Darmstadt, Germany) for another 14 days. For siRNA-mediated gene knockdown, BGC823 and SGC7901 cells were transfected with negative control siRNA or IGFBP3-targeted siRNA (Qiagen, Hilden, Germany), HGC27 cells were transfected with negative control siRNA or HoxD10-targeted siRNA (Genepharma, Shanghai, China) using Lipofectamine RNAiMAX Reagent (Invitrogen). 

### Luciferase activity assay

1.0×10^5^ BGC823 and SGC7901 cells were seeded in 24-well plates for 24h, then transfected with 0.4μg pcDNA3.1 empty vector or pcDNA3.1-HoxD10, 0.04μg pGL3-basic (promoter) empty vector or pGL3-pIGFBP3(HBS)-basic(promoter), and 0.004μg pRL-TK vector (Promega) containing reference control renilla using Fugene HD [18]. Luciferase activity was analyzed by the dual-luciferase reporter assay system after 48h according to the manufacturer's protocols (Promega).

### Chromatin immunoprecipitation (ChIP)

In stable HoxD10 overexpressed BGC823 and SGC7901 cell lines, the chromatin was immunoprecipitated with HoxD10 monoclonal antibody (Rabbit, Santa Cruz Biotechnology Inc., Santa Cruz, USA) [19] or normal rabbit IgG (negative control) according to the Simple ChIP Enzymatic Chromatin IP Kit (Cell Signaling Technology Inc., Danvers, USA). Precipitated DNAs or 2% Input samples with genomic DNA from HEK-293T wild cells were amplified with Taq DNA Polymerase (TaKaRa, Otsu, Japan) [20,21]. Four primers encompassing HBSs in the different sites of IGFBP3 promoter were designed as follows: A1 （-2251~ -2073 bp, for HBS1 and 2,), 5’-CATTCGGCACTGAACAAG-3’ and 5’-AGCCTGGACTGACCACT-3’; A2 （-1765~ -1633 bp, for HBS3,), 5’-CATAAGAAAATGACGGTGCT-3’ and 5’-TGGAAAGATCAATTTCGTTCC-3’; A3 （-1470 ~ -1364bp, for HBS4,), 5’-AAGATTAACTTCACCCAAGGC-3’ and 5’-AGGTGGATAGGTGACTTG-3’; A4 （-1153~ -877bp, for HBS5,), 5’-GCCGACAGGAGTTACAG-3’ and 5’-TGTTCTTCTTGTCTTGGGTA-3’.

### Reverse transcription-PCR

Total RNA was isolated with Trizol reagent (Cwbiotech, Beijing, China). Reverse transcription (RT)-PCR were determined with M-MLV reverse transcriptase (TaKaRa) and Taq DNA Polymerase. The following primers were used for amplification: 

GAPDH, 5’- GAAGGTGAAGGTCGGAGT-3’ and 5’-GAAGATGGTGATGGGATTTC-3’; HOXD10, 5’-AAAGTCTCCCAGGTGGAGAG-3’ and 5’-TGCTGGTTGGTGTATCAGAC-3’; IGFBP3, 5’-CCAAGCGGGAGACAGAAT-3’ and 5’-CTTTGGAAGGGCGACACT-3’; 

MMP2, 5’-ACGACCGCGACAAGAAGTAT-3’ and 5’-ATTTGTTGCCCAGGAAAGTG-3’; 

MMP7, 5’-GGTATGGGACATTCCTCTGA-3’ and 5’-TGTTCTGCCTGAAGTTTCTATT-3’; 

MMP9, 5’-TCGAACTTTGACAGCGACAAG-3’ and 5’-GCACTGAGGAATGATCTAAGC-3’; 

MMP14, 5’-GGCGGGTGAGGAATAAC-3’ and 5’-AGCATCAATCTTGTCGGTAG-3’;

tissue inhibitor of metalloproteinase-1 (TIMP1), 5’-GCTTCTGGCATCCTGTTGTT-3’ and 5’-GGTATAAGGTGGTCTGGTTGACT-3’; 

TIMP2, 5’-AAGCGGTCAGTGAGAAGGAA-3’ and 5’-TCTCAGGCCCTTTGAACATC-3’; 

uPA, 5’-CTCATCCTACACAAGGACTAC-3’ and 5’-CAGGCAGATGGTCTGTATAGT-3’; uPAR, 5’-ATTCCCGAAGCCGTTAC-3’ and 5’-GTTGCATTTGGTGGTGTT-3’. 

### Western blotting

Total proteins were extracted using RIPA lysis buffer (Beyotime, Haimen, China). Triton X-100 lysis buffer was used to extract soluble E-cadherin and ß-catenin, SDS lysis buffer was used to extract insoluble E-cadherin/ß-catenin complex [22]. Lysates were resolved on SDS-PAGE gel and transferred to PVDF membranes (Millipore, Bedford, USA). The blots were probed with HoxD10 (1:1000, Santa Cruz Biotechnology Inc.), IGFBP3 (1:500, Santa Cruz Biotechnology Inc.), E-cadherin (1:1000, Cell Signaling Technology Inc.), ß-catenin (1:1000, Cell Signaling Technology Inc.) or GAPDH (1:2500, Cwbiotech) antibodies. The blots were visualized using a chemiluminescence with Las-4000 Imaging System (Fujifilm, Tokyo, Japan). The relative densities of proteins were quantified with Image J. software and normalized to GAPDH [20].

### Wound-healing assay

BGC823 and SGC7901 cells were transfected with IGFBP3 siRNA or control negative siRNA in six-well plates and then scratched with a p10 pipette tip to create a gap. The wells were rinsed with PBS to remove displaced cells and fresh media (1% FBS for BGC823 and serum free for SGC7901) was added. Three randomized images of the scratched areas were taken (×40 magnification) over 0h, 12h and 24h [23].

### Transwell migration and invasion assays

Cell migration and invasion were assessed by modified Boyden transwell chambers (Corning Inc., Corning, USA), coated with (for invasion) or without (for migration) matrigel (BD Biosciences, Franklin Lakes, USA). siRNA transfection and starvation cells were plated to the upper chamber in culture medium containing 1% FBS (BGC823) or no FBS (SGC7901), medium containing 15% FBS was added to the lower chamber, cells in the upper chamber were carefully removed after incubation for 16h (for migration) or 36h (for invasion). Migrated cells were stained with 0.5μg/ml DAPI Staining Solution (Roche, Penzberg, Germany). The cell numbers were randomly counted in five fields (×400 magnification) [24]. Invaded cells were incubated with Cell Stain Solution (Millipore) and photographed (×200). The dye mixture was washed by Extraction Buffer and transferred to a 96-well for colorimetric measurement at 560nm in a microplate reader (Thermo, Boston, USA) [25,26].

### Patients and specimens

86 surgery patients of gastric adenocarcinoma were enrolled from May 2007 to February 2008 after signing the informed consent. This study was approved by Clinical Research Ethics Committee of Sir Run Run Shaw Hospital of Zhejiang University. Matched tumor tissues and adjacent tumor-free tissues were obtained. Patients’ clinicopathological data including gender, age, TNM stages and pathological grades were retrieved from medical records. Follow-up was performed at an 1-year interval after the surgery, a medical history was recorded when the patient came for subsequent visit. The follow-up cutoff time was August 2012, and the median follow-up time was 35 months (range, 1-63 month). 

### Tissue microarray and immunohistochemical staining

Cores measuring 1.5mm in the greatest dimension were punched from non-necrotic areas of matched tumor tissues and adjacent tumor-free tissues. Tissue microarray slides containing 4μm thick microarray sections were constructed using standard techniques (in collaboration with Shanghai Superchip Company, Ltd., Shanghai, China). Slides were incubated with IGFBP3 antibody (1:100, Santa Cruz Biotechnology Inc.) overnight at 4°C, and then incubated with the Envision-plus detection system (EnVision™+/HRP/Rb, Dako, Copenhagen, Danmark). The sections were developed in 3,3′-diaminobenzidine solution under microscopic observation and counterstained with hematoxylin [27]. Tissues of advanced breast cancer were stained as the positive control [28]. The proportion of positive cells in each specimen was quantified under microscope and classified into four groups. 0: 0-5% positive cells; 1: 6% to 50% positive cells; 2: 51% to 75% positive cells and 3: 76-100% positive cells. The intensity of IGFBP3 staining was graded as follows: no staining = 0; weak staining = 1; moderate staining = 2; dense staining = 3. The score of the intensity plus the proportion of positive staining was defined as IGFBP3 staining score. A score of 0-3 was considered as low expression and 4-9 as high expression. 

### Statistical analysis

The differential expression of IGFBP3 between tumorous tissue and non-tumorous tissue was determined by Mann-Whitney U-test. Chi-square test was used to analyze the relationships between clinicopathological features and IGFBP3 staining scores. The probability of overall survival was calculated with the Kaplan-Meier method and the difference between curves was evaluated with the Log-rank test. A cutoff of p<0.05 was considered to be statistically significant.

## Results

### HoxD10 upregulates the expression of IGFBP3

**Figure 1:**
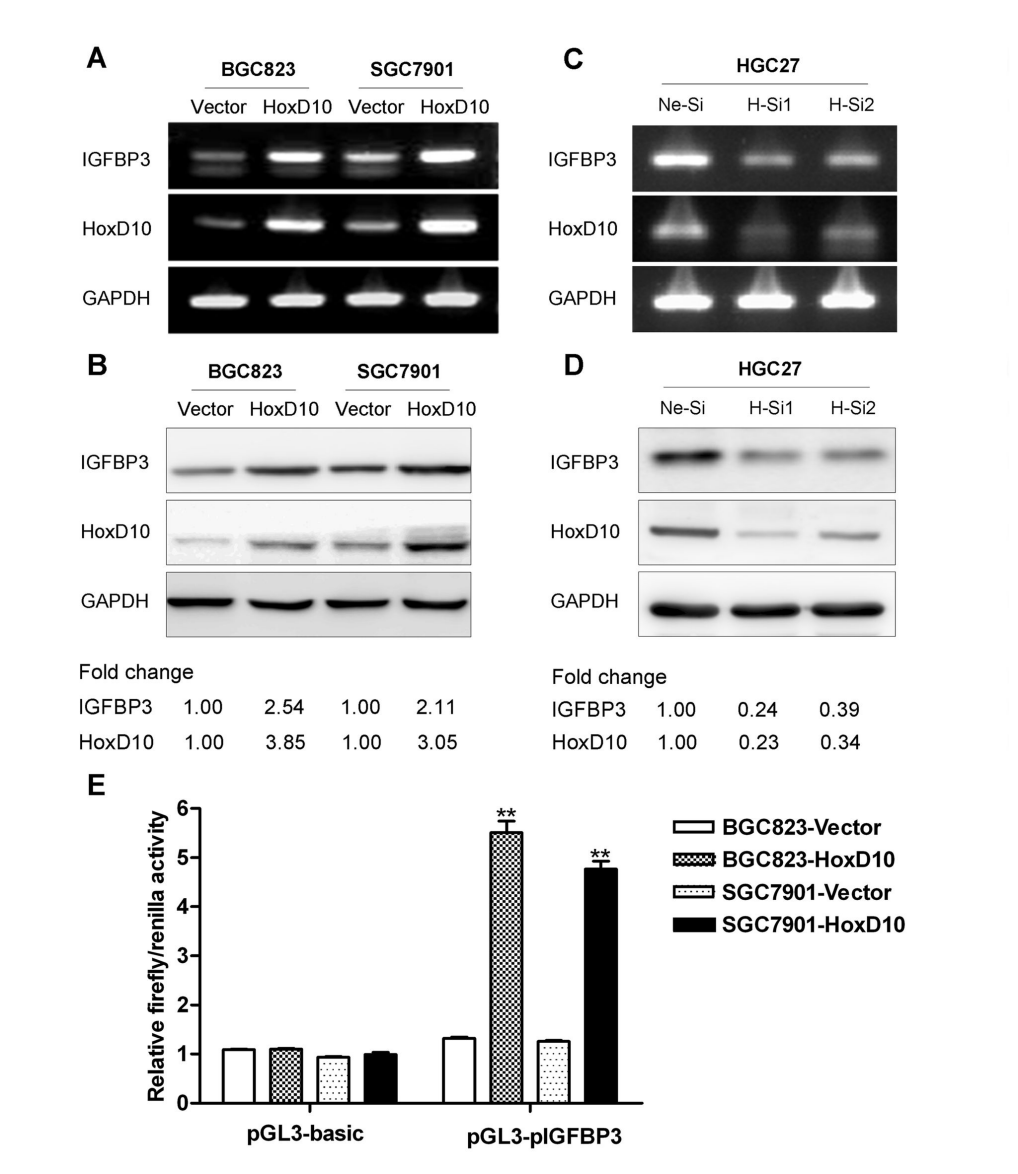
The expression of IGFBP3 is regulated by HoxD10 in human gastric cancer cells. The expression of IGFBP3 was detected by conventional RT-PCR (**A**, **C**) and Western blotting (**B**, **D**) after transfected with pcDNA3.1 empty vector or pcDNA3.1-HoxD10 in BGC823 and SGC7901 cells, and negative control siRNA (Ne-Si) or two of HoxD10 siRNA (H-Si1 and H-Si2) in HGC27 cells. GAPDH was used as internal control. Densitometry values are expressed as fold change compared with pCDNA3.1 vector or negative control siRNA values normalized to 1. (**E**) BGC823 and SGC7901 were co-transfected with pcDNA3.1 empty vector or pcDNA3.1-HoxD10, pGL3-basic vector or pGL3-(pIGFBP3)-basic and pRL-TK vector. Relative firefly activity was expressed normalized to renilla activity in pRL-TK vector. All experiments were performed in triplicate. ** indicates of p<0.01.

Genome-wide analysis revealed that multiple genes including IGFBP3 were regulated by HoxD10 in gastric cancer cells in our previous study (4). To determine whether HoxD10 could transcriptionally regulate the expression of IGFBP3 in gastric cancer cells, we observed the expression level of IGFBP3 after stably introducing HoxD10 gene into BGC823 and SGC7901 cells, or transiently knocking down of HoxD10 in HGC27 cells. RT-PCR and Western blotting results consistently showed that the expression of IGFBP3 was significantly upregulated in HoxD10 overexpressed cells (Figure **1A and 1B**), while downregulated after silencing of HoxD10 in HGC27 cells (Figure **1C and 1D**).

A 2.3 kb sequence (-2282 to +56 bp) at the upstream region of IGFBP3 was cloned into pGL3-basic vector and used to evaluate luciferase activity by dual-luciferase reporter assays. Results demonstrated that the IGFBP3 promoter activity in BGC823 and SGC7901 cells was enhanced by 4.2 and 3.8 folds respectively after co-transfected with pcDNA3.1-HoxD10 relative to pcDNA3.1 vector transfectants (p<0.01, Figure **1E**). Taken together, these data suggested that HoxD10 could transcriptionally upregulate the expression of IGFBP3 in gastric cancer cells.

### Identification of novel regulatory regions in the IGFBP3 promoter responsible for HoxD10

**Figure 2:**
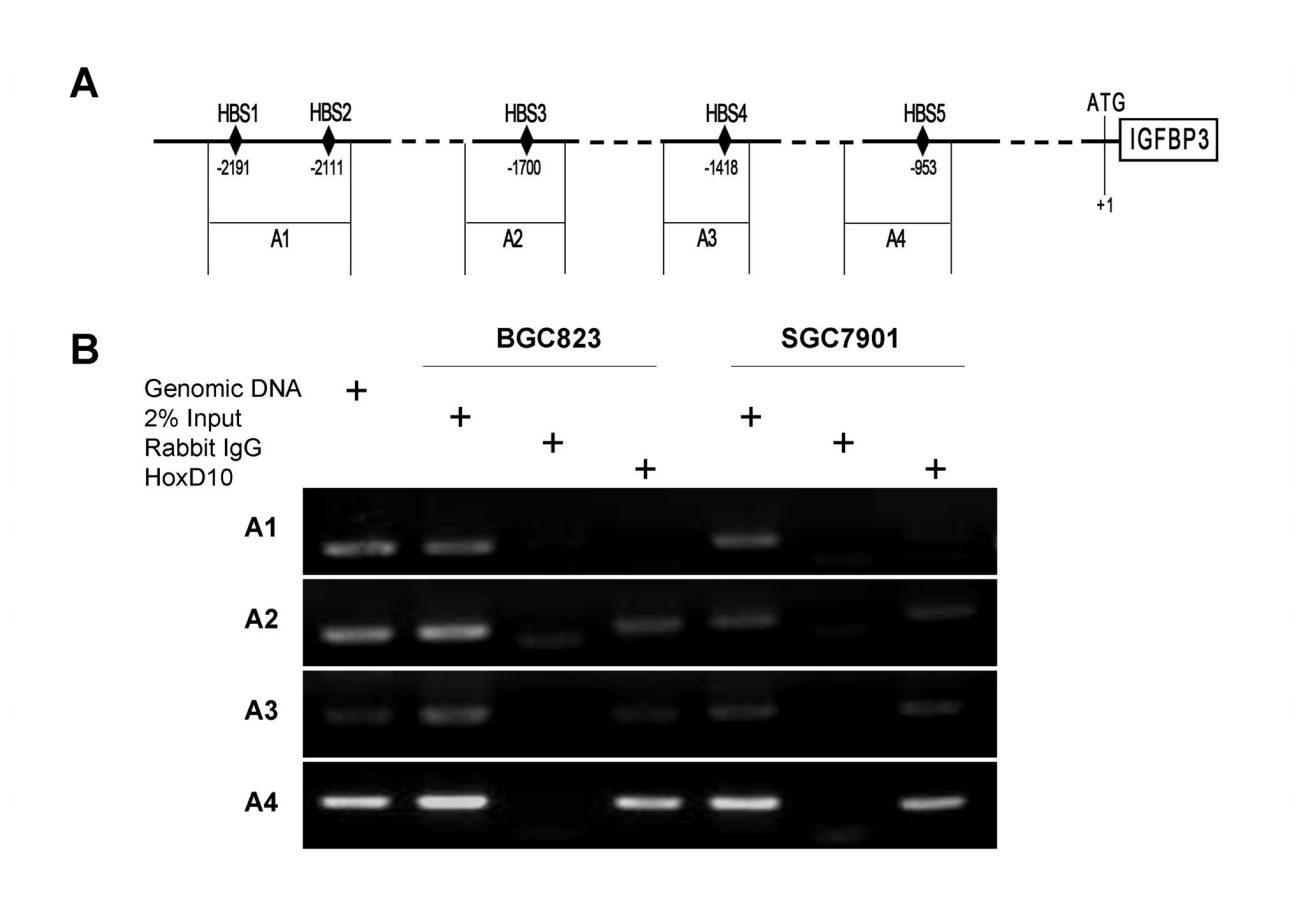
HoxD10 binds to the promoter region of IGFBP3 gene. (**A**) Schematic representation of IGFBP3 promoter with the potential binding sites for HoxD10 (HBS1-5) and four fragments (A1-A4) spanning five HBSs at the IGFBP3 promoter region were designed for PCR analysis in ChIP assays (A1-A4). (**B**) HoxD10 overexpressed BGC823 and SGC7901 cells were cross-linked by formaldehyde and lysed. Cell lysates were subjected to immunoprecipitation with either a rabbit antibody to HoxD10 or normal rabbit IgG. Genomic DNA and 2% input DNA were used as positive control.

We next analyzed the potential HoxD10 binding sites in the IGFBP3 promoter. The 2.3 kb upstream sequence of IGFBP3 gene was inputted into PROMO (http://alggen.lsi.upc.es/), a program for the prediction of transcription factor binding sites in a single sequence or in a group of related sequences (17), and 5 potential HoxD10 binding sites (HBS1~5) were predicted (Table **S1**). As shown in Figure **2A**, these five HBSs were localized at -2191~ -2182bp (HBS1), -2111~ -2102bp (HBS2), -1700 ~ -1691bp (HBS3) , -1418 ~ -1409bp (HBS4) and -953 ~ -944bp (HBS5) respectively. In stable HoxD10 overexpressed BGC823 and SGC7901 cells, binding regions at IGFBP3 promoter were investigated by ChIP assays. We identified that the chromatin precipitated by HoxD10 specific antibody was amplified using A2, A3 and A4 primers, which encompass HBS3, HBS4 and HBS5 respectively, while no amplification was observed with A1 primers encompassing HBS1 and 2 (Figure **2B**). 

**Figure 3:**
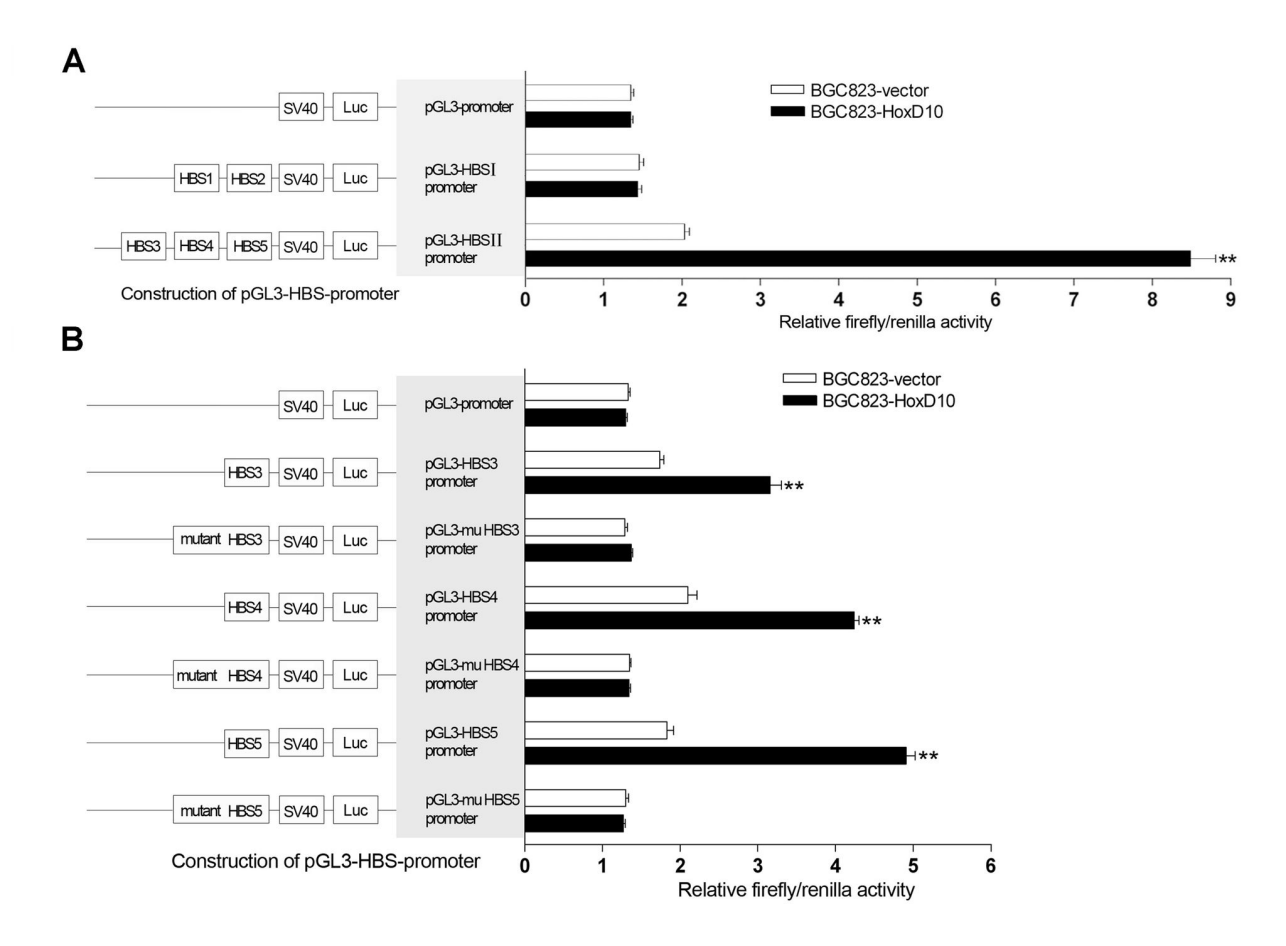
Identification of the regulatory regions of IGFBP3 which are responsible by HoxD10 in BGC823 cells. (**A**) BGC823 cells were transfected with pcDNA3.1 empty vector or pcDNA3.1-HoxD10, pGL3-promoter vector or pGL3-HBSI(II)-promoter and pRL-TK vector. (**B**) Different oligonucleotides which contain wild or point mutant sequences of HBS3, HBS4 and HBS5 were cloned into pGL3-HBS-promoter. Relative firefly activity was expressed normalized to renilla activity in pRL-TK vector. All experiments were performed in triplicate. ** indicates of p<0.01.

To gain further into the regulatory segments of IGFBP3 promoter by HoxD10, we cloned 2 different DNA fragments, which encompass HBSI (HBS1 and 2 ) and HBSII (HBS3, 4 and 5), respectively, into SV40 promoter luciferase reporters. Results showed that co-transfected with HoxD10, HBSII enhanced SV40 promoter activity by 4.0-fold when compared with those empty vector transfectants in BGC823 cells (P<0.01, Figure **3A**). In contrast, HBSI showed no significant effect on the SV40 promoter activity (Figure **3A**). Similar results were revealed in another independent SGC7901 cells, the SV40 promoter activity changes were 3.3 and 0.9 fold by HBSII and HBSI, respectively (**Figure S1A in File S1**). 

HBS3, 4 and 5 shared common binding element “TTAT”, while HBS1 or HBS2 have none of these elements. We hypothesis that HoxD10 directly binds to the promoter of IGFBP3 at “TTAT” sites. To confirm this, we synthesized 10bp oligonucleotides which contain the sequences of HBS3, HBS4 and HBS5 respectively. As shown in Figure 3B, The SV40 promoter activities were enhanced by 1.8, 2.0, and 2.7 folds respectively when co-transfected with HoxD10 plasmid in BGC823 cells, while point mutants of HBS3, HBS4 and HBS5 showed no significant changes. We reproduced similar results in SGC7901 cells (**Figure S1B in File S1**). 

 Collectively, above results indicate that IGFBP3 is a direct target of HoxD10 in gastric cancer cells. We identified three functional bindings sites between -1727~ -943bp in the upstream of IGFBP3 gene responsible for HoxD10. HoxD10 could directly bind the promoter of IGFBP3 potentially through Hox binding element “TTAT”. 

### IGFBP3 is downregulated in gastric cancer and related to the patients’ prognosis

**Figure 4:**
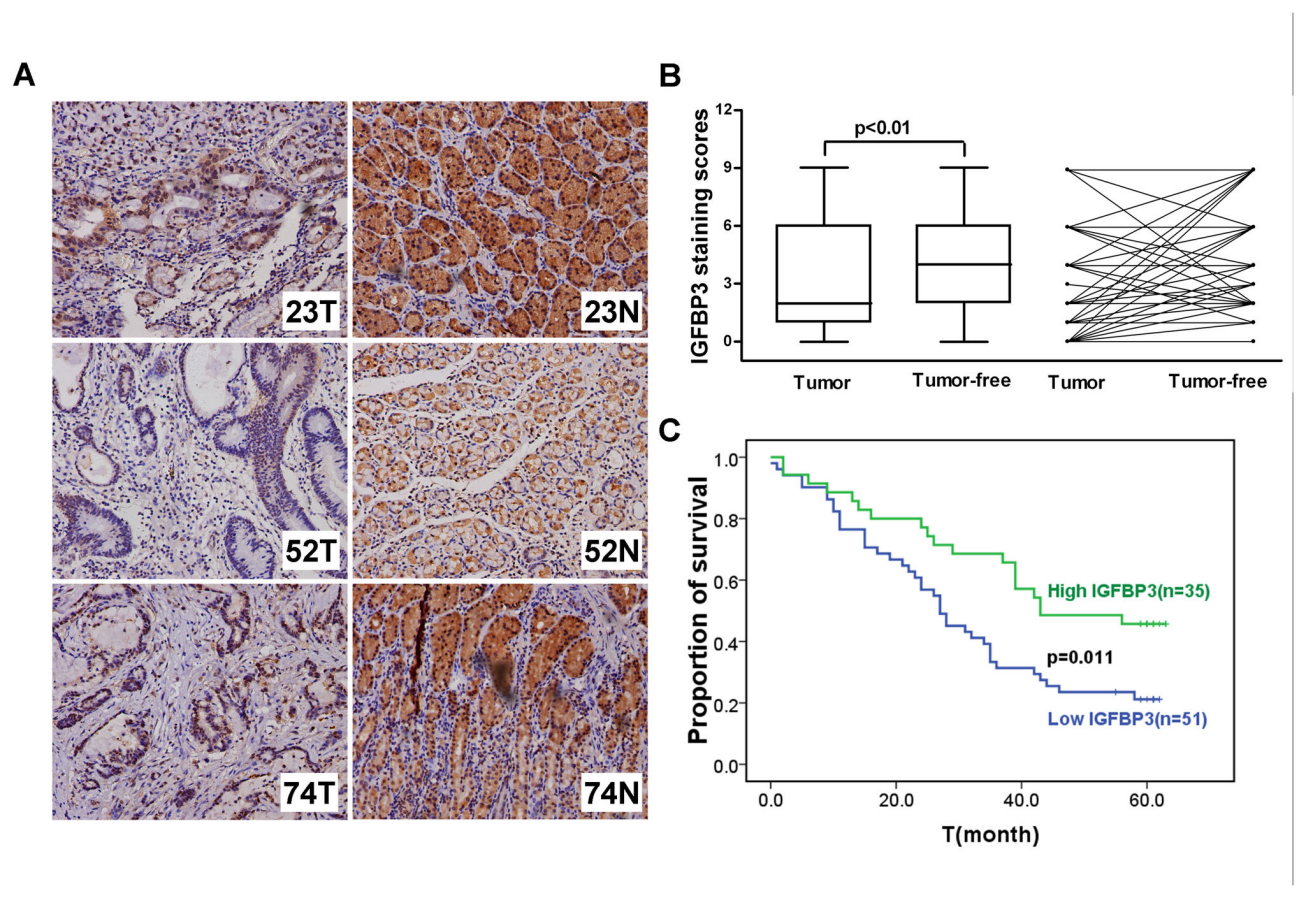
The expression of IGFBP3 is downregulated in gastric tumor tissues and related to the survival of patients. (**A**) The expression levels of IGFBP3 were detected by immunochemistry analysis in 86 pairs of gastric tumor and matched adjacent normal tissues. Representative photos were shown in 3 paired tumor (T) and normal (N) tissues. (**B**) The comparison of staining scores of IGFBP3 between gastric tumor and adjacent tumor free tissues. (**C**) Survival curves were plotted based on the Kaplan-Meier survival analysis. The expression level of IGFBP3 was used as the variate to separate two lines.

To determine the expression pattern of IGFBP3 in gastric cancer, 86-pair surgical specimens of human gastric cancer and according adjacent noncancerous gastric tissues were examined. The intensity of IGFBP3 staining was scored as 0 (negative), 1 (weak), 2 (moderate), or 3 (dense), as determined independently by two pathologists (**Figure S2 in File S1**). Results showed that the expression level of IGFBP3 in tumor tissues was significantly lower than that in adjacent tumor-free tissues (p<0.001, Figure **4A and 4B**). Furthermore, IGFBP3 expression was evaluated with regards to the clinicopathological characteristics of the patients. IGFBP3 expression was significantly lower in gastric tumor with lymph node metastasis (p=0.045). In addition, high IGFBP3 expression in small-size tumors (T1+T2, 8/12) was more frequently observed relative to that in large ones (T3+T4, 36/74), though with no significant difference (p=0.062). There was also no significant correlation between the expression of IGFBP3 and clinicopathological features including gender, age and pathological grade (Table **1**). 

**Table 1:** Gender, age and clinicopathological characteristics and immunohistochemistry results of gastric tumor tissue samples.

		IGFBP3 immunostaining intensity		
Clinical classification	Total number	Low(number)	High(number)	p value
Gender				0.812
Male	63	38	25	
Female	23	13	10	
Age(years)				0.644
<60	27	15	12	
≥60	59	36	23	
T				0.062
T1+T2	12	4	8	
T3+T4	74	47	27	
N				0.045
N0+N1	35	16	19	
N2+N3	51	35	16	
M				0.267
M0	83	48	35	
M1	3	3	0	
Histological grade				0.331
I+II	24	12	12	
III	62	39	23	

We further analyzed the correlation between the expression of IGFBP3 and the overall survival of gastric cancer patients. Higher expression of IGFBP3 was associated with longer survival time in the 5 years follow-up (p=0.011, Kaplan-Meier survival Log-rank test) (Figure **4C**).

### Knockdown of IGFBP3 gene promotes gastric cancer cell migration and invasion

**Figure 5:**
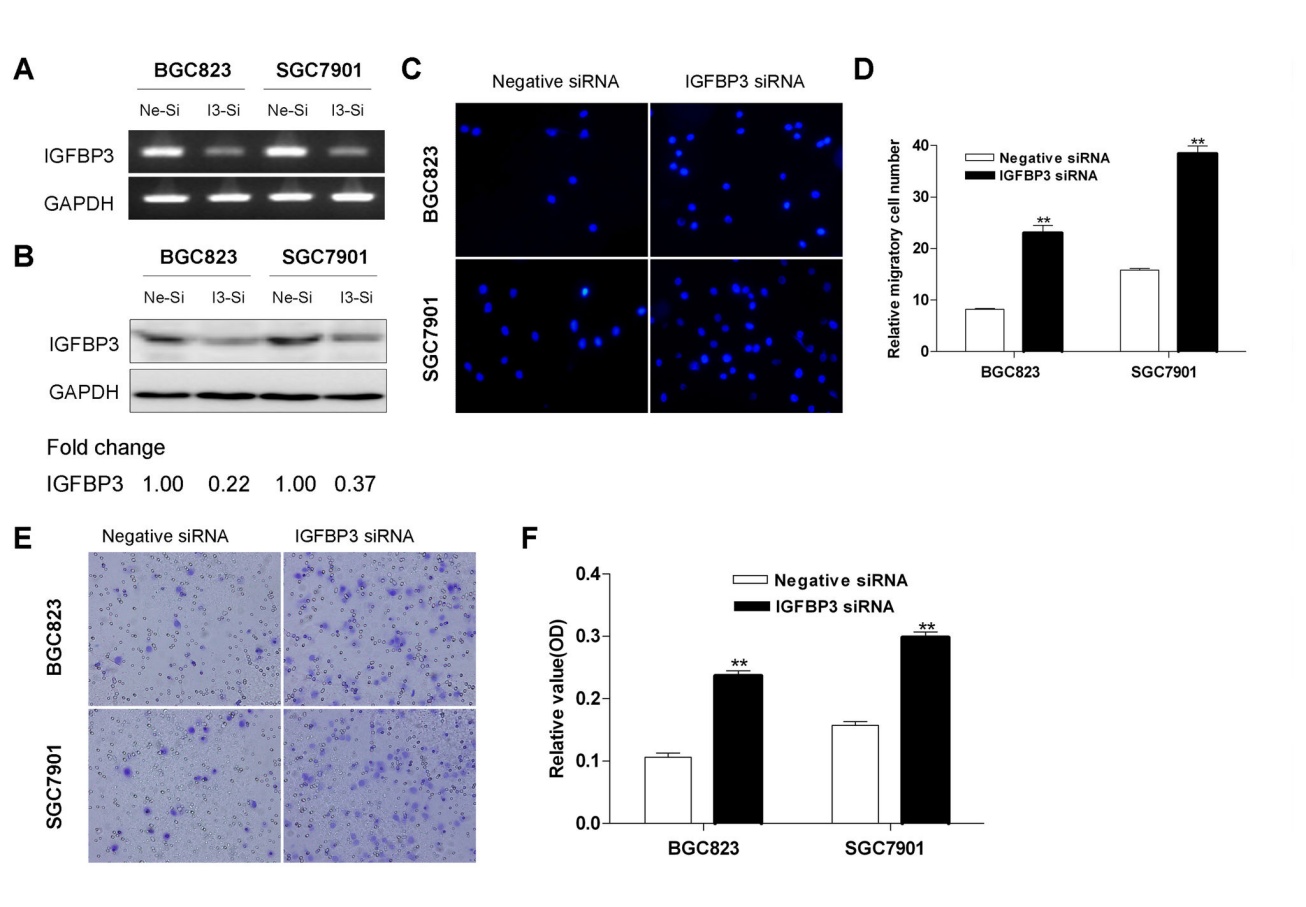
IGFBP3 inhibits the migration and invasion of gastric cancer cells. The expression of IGFBP3 in BGC823 and SGC7901 cells were detected by conventional RT-PCR (**A**) and Western blotting (**B**) after transfected with negative control siRNA (Ne-si) or IGFBP3 siRNA (I3-si). Densitometry values are expressed as fold change compared with negative control siRNA values normalized to 1. (**C**) Cell migration was assessed by modified Boyden transwell chambers assays. After incubation for 16h, cells that migrated to the bottom of the membrane were stained with DAPI. (**D**) The mean number of visible migratory cells was counted in five random high power fields. These experiments were performed in triplicate. ** indicates of p<0.01. (**E**) BD Matrigel coated chambers were used to assess cell invasion. Invaded cells on the bottom of the membrane were stained with cell stain solution. (**F**) Invaded cells were washed by Extraction Buffer and detected on a microplate reader (560 nm). ** indicates of p<0.01.

To evaluate the functional role of IGFBP3 in gastric cancer, we investigated the cell migration and invasion after knockdowning IGFBP3 in vitro. IGFBP3 was selectively knockdowned by RNA interference in BGC823 and SGC7901 cells (Figure **5A and 5B**), which had a relative high level of endogenous IGFBP3 in a panel of gastric cell lines (**Figure S3 in File S1**). Results showed that silencing expression of IGFBP3 significantly enhanced cell migration both in transwell migration assays (Figure **5C and 5D**) and scratch test (**Figure S4 in File S1**), when compared to that of negative control siRNA. In addition, knockdowning IGFBP3 displayed a significantly higher activity of cellular invasion in the transwell assays (Figure **5E and 5F**). 

### IGFBP3 modulates the expressions of MMP14, uPA and uPAR

**Figure 6:**
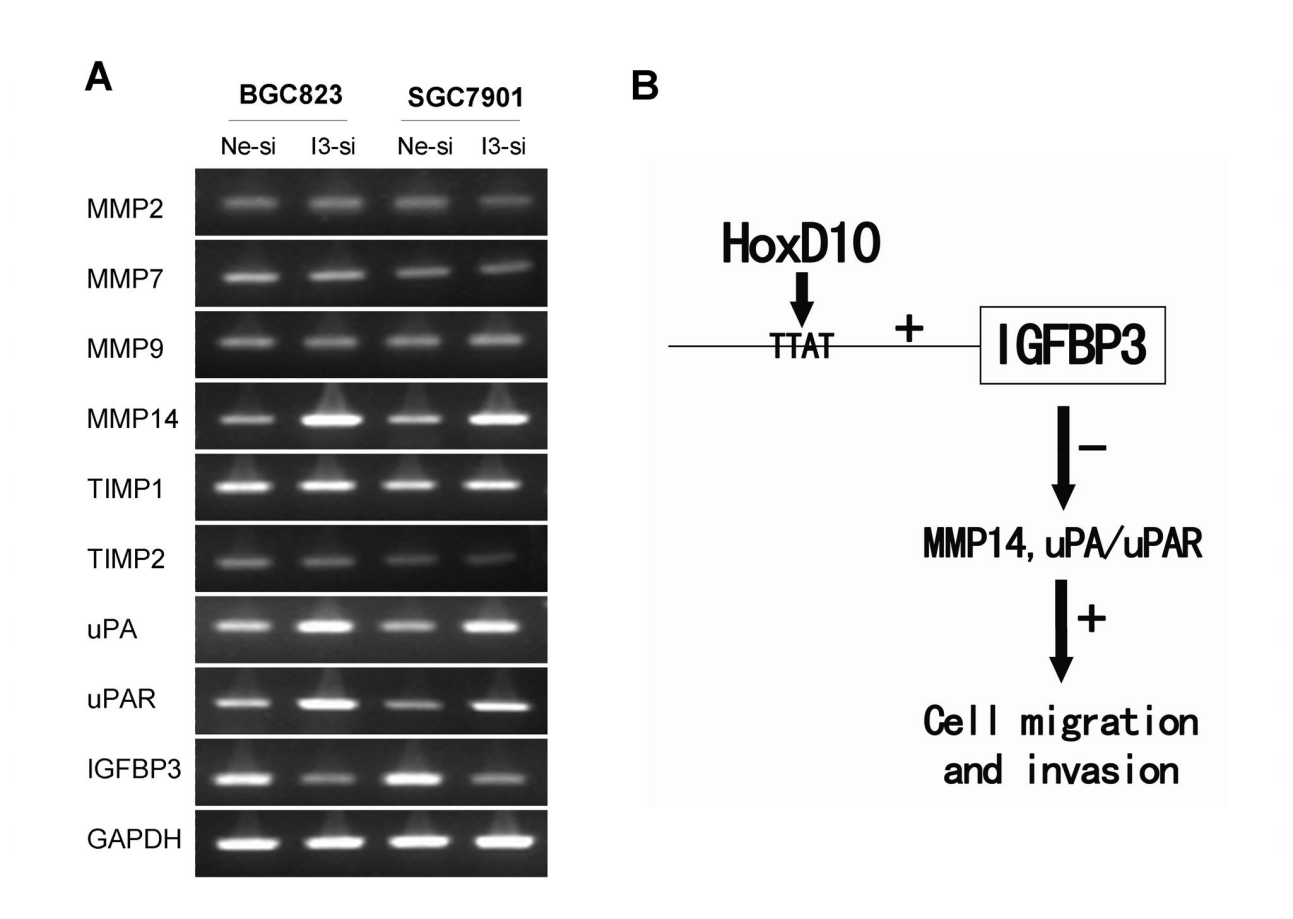
IGFBP3 upregulates the expression of MMP14, uPA and uPAR. (**A**) MMPs (2,7,9,14), TIMPs (1,2), uPA and uPAR in BGC823 and SGC7901 cells were detected by conventional RT-PCR after transfected with negative control siRNA (Ne-si) or IGFBP3 siRNA (I3-si). (**B**) Systemic understanding of pathways for HoxD10-IGFBP3 regulation of gastric cancer cell migration and invasion.

To figure out the possible mechanisms of how IGFBP3 regulate the invasiveness of gastric cancer cells, we examined the mRNA levels of MMP2/MMP7/MMP9/MMP14, TIMP1/TIMP2, uPA and its receptor uPAR, all of which are invasion-related factors, especially in gastric cancer. After transiently transfected with IGFBP3 siRNA for 72h in BGC823 and SGC7901 cells, the mRNA expression levels of MMP14, uPA and uPAR increased, while no significant change of MMP2, MMP7, MMP9 or TIMP1/TIMP2 was observed (Figure **6A**). E-cadherin, ß-catenin and E-cadherin/ß-catenin complex were extracted using different lysates, their levels had no meaningful change after silencing IGFBP3 in BGC823 cells (**Figure S5 in File S1**). These results indicated that IGFBP3 inhibits the invasion of gastric cancer cells through, at least in part, modulating the expressions of MMP14, uPA and uPAR (Figure **6B**).

## Discussion

Hox superfamily are a group of important transcription factors which could regulate the proliferation and differentiation of embronic cells [29,30]. In addition, the aberrant expression of Hox also plays critical roles in the progression of several types of cancers [31]. Hox proteins have a large downstream regulatory network and exert their functions mainly through these target genes [5]. Genome-wide analysis has been carried out to screen the downstream genes of HoxD10 during spinal cord development and in gastric cancer cells [4,32]. We and others have revealed one common target gene IGFBP3, which has been reported to serve as a tumor suppressor [10]. 

Our studies provided first evidence to show that HoxD10 directly binds the IGFBP3 promoter to activate the expression of IGFBP3 in gastric cancer cells. HoxD10 protein could be recruited to the specific promoter region of IGFBP3 gene, indicating that HoxD10 could directly bind to IGFBP3 gene. The luciferase activity with wild, while not mutant, HBS3, HBS4 or HBS5 was significantly enhanced by overexpression of HoxD10, suggesting that HBS3 (TCTTTTTATT), HBS4 (ATTTGCTATT) and HBS5 (CTTTATTATT) were three functional HoxD10 binding sites at the promoter region of IGFBP3. As expected, the expression level of IGFBP3 mRNA and protein were upregulated by forced expression of HoxD10 in different gastric cancer cell lines [4]. Studies have shown that Hox proteins have some core consensus binding elements, including TTAT, TAAT, and TTAC [7]. Point mutation with “TTAT” to “TTCT” or “TCTT” to “TATT” in the promoter region of IGFBP3 in the present study indicate that “TTAT” or “TATT” are important binding sites for HoxD10. Hox proteins could directly regulate the transcription process of downstream target genes as monomers or homodimers [33]. Since DNA-binding competence of Hox proteins itself is relatively low [34], interactions with cofactors like extradenticle (Exd)/Pbx and Homothorax (Hth)/Meis proteins as heterodimers or heterotrimers are critical to the target selectivity of Hox proteins [33,35]. Other transcription factors including PTEN and p53 could regulate the expression of IGFBP3 at the transcriptional level in gastric and colonic carcinoma cells [36,37]. The exact HoxD10 regulatory mechanisms and the cofactors involved in the regulation of IGFBP3 need to be clarified in future. 

The expression of IGFBP3 was downregulated in 86 gastric adenocarcinomas tissues relative to their adjacent normal tissues, and low expression of IGFBP3 was also correlated with advanced lymph node metastasis, distant metastasis and poor overall survival. Our findings were consistent with previous report that well or moderate-differentiated gastric adenocarcinomas had significantly higher percentage of IGFBP3 staining in tumor tissues than those in poor-differentiated ones [38]. In addition, hypermethylation in the promoter of IGFBP3 was frequently detected in gastric tumor tissues [16,39,40]. Functional IGFBP3 SNPs (rs2854744 and rs2960436) determining high IGFBP3 circulating levels were associated with favorable prognosis of patients with advanced gastric cancer receiving palliative chemotherapy [41]. However, another study showed the quite contrary results, suggesting that the expression of IGFBP3 was higher in tumor samples than that in normal mucosa (54 tumor samples and 20 adjacent normal samples, not matched), and positive expression of IGFBP3 was associated with advanced histological grade, lymph node metastasis and distant metastasis without significant difference [42]. The possibility of intratumor heterogeneity and enrolled criteria may induce different results in different clinical studies. Large populations and prospective studies were allowed to evaluate the potential utility of IGFBP3 as a biomarker of the progression of gastric cancer.

To further identify the role of IGFBP3 in the metastasis of gastric cancer, we evaluated its role in modulating the migration and invasion of gastric cancer cells. It turned out that silencing expression of IGFBP3 resulted in rapid healing of the scratch wound and increased number of cells migrating through the transwell membrane whether coated with or without matrigel. Previous studies mostly focused on the function of IGFBP3 in cell proliferation and apoptosis. Only several studies reported it was related with metastasis in EC, prostate cancer and HNSCC [13-15]. Silencing expression of IGFBP3 could induce cell migration, invasion and metastasis in EC [13]. IGFBP3 knockout male mice had a higher incidence of lung metastases and the invasiveness of primary prostatic tumor cells was increased over 3-fold when compared with that of wild ones [14]. Adenoviral and recombinant IGFBP3 inhibited vascularization and angiogenesis-stimulating activities of HNSCC by suppressing the production of vascular endothelial growth factor (VEGF) [15]. Migration and invasion are facilitated by a number of factors capable of inducing epithelial-mesenchymal transition (EMT), like E-cadherin, ß-catenin and factors degrading the extracellular matrix, including MMPs, uPA and so on [43]. In EC cells, siRNA targeting IGFBP3 increases the expression of MMP2 [13]. In this study, we detected those metastasis relevant factors, including MMP7, MMP9 and MMP14, which are particularly important in gastric cancer [44]. TIMP1 and TIMP2, which could complex with MMPs and irreversibly inactivate the expression of MMPs, were also detected. The mRNA levels of MMP14, uPA and uPAR were uprequlated after knockdown of IGFBP3 in gastric cancer cells. Interestingly, as one of upstream regulator of IGFBP3, HoxD10 inhibits the migration and invasion of glioma and gastric cancer cells partly by modulating the expression of MMP14 and uPAR, which are both tumor invasion promoting factors [26,45]. 

In summary, we identified IGFBP3 as a transcriptional target of HoxD10. IGFBP3 inhibits the migration and invasion of gastric cancer cells, at least in part, through the MMP14 and uPA/uPAR pathways (Figure **6B**). The expression of IGFBP3 is related to less metastasis and favors the survival of gastric cancer patients.

## Supporting Information

File S1 **Supplementary Figure.** **Figure S1. Identification of the regulatory regions of IGFBP3 which are responsible by HoxD10 in SGC7901cells.** (A) SGC7901 cells were transfected with pcDNA3.1 empty vector or pcDNA3.1-HoxD10, PGL3-promoter vector or PGL3-HBSI (II)-promoter and pRL-TK vector. (B) Different oligonucleotides which contain the wild or point mutant sequences of HBS3, HBS4 and HBS5 were cloned into PGL3-HBS-promoter. Relative firefly activity was expressed normalized to renilla activity in pRL-TK vector. All experiments were performed in triplicate. ** indicates of p<0.01. **Figure S2. Staining density of IGFBP3 in gastric tissues.** The expression levels of IGFBP3 were detected by immunochemistry analysis in gastric tumor (1-4) and adjacent tumor free tissues (6-8), as well as breast tumor tissue (5, positive control). The intensities of IGFBP3 staining were graded as follows: no staining = 0; weak staining = 1; moderate staining = 2; dense staining = 3. Tissue 1 was scored as “0”, 2 and 6 scored as “1”, 3 and 7 scored as “2”, 4 and 8 scored as “3”. **Figure S3. Expression of IGFBP3 in a penal of human gastric cancer cell lines.** Conventional RT-PCR (A) and Western blotting (B) were used to detect expression levels of IGFBP3 in 8 human gastric cancer cell lines (AGS/MKN28/MKN45/NCI-N87/BGC823/HGC27/MGC802/SGC7901) and human gastric epithelial immortalized GES-1 cells. **Figure S4. IGFBP3 inhibits gastric cancer cell migration (scratch test).** Cell migration was detected by wound-healing assay in BGC823 and SGC7901 cells. The wound in the area of cells was curved when the cell migrated for 12h and 24 h after transfected with negative control siRNA (Ne-si) or IGFBP3 siRNA (I3-si). Representative images were taken with 40 magnification power from triple experiments. **Figure S5. Effects of IGFBP3 on E-cadherin and β-catenin in BGC823 cells.** Total, soluble and insoluble E-cadherin and β-catenin in BGC823 cells were detected by Western blotting after transfected with negative control siRNA (Ne-si) or IGFBP3 siRNA (I3-si).
(PDF)

Table S1 **Possible HoxD10 binding sites at the promoter region of IGFBP3.**
(DOC)
