# Supplementary material for: IGFBP3, a Transcriptional Target of Homeobox D10, Is Correlated with the Prognosis of Gastric Cancer
Source: PLoS One. 2013 Dec 27;8(12):e81423. doi: 10.1371/journal.pone.0081423 (PMC3873913; doi:10.1371/journal.pone.0081423)
Supplement: File S1 — Supplementary Figure. Figure S1. Identification of the regulatory regions of IGFBP3 which are responsible by HoxD10 in SGC7901cells. (A) SGC7901 cells were transfected with pcDNA3.1 empty vector or pcDNA3.1-HoxD10, PGL3-promoter vector or PGL3-HBSI (II)-promoter and pRL-TK vector. (B) Different oligonucleotides which contain the wild or point mutant sequences of HBS3, HBS4 and HBS5 were cloned into PGL3-HBS-promoter. Relative firefly activity was expressed normalized to renilla activity in pRL-TK vector. All experiments were performed in triplicate. ** indicates of p<0.01. Figure S2. Staining density of IGFBP3 in gastric tissues. The expression levels of IGFBP3 were detected by immunochemistry analysis in gastric tumor (1-4) and adjacent tumor free tissues (6-8), as well as breast tumor tissue (5, positive control). The intensities of IGFBP3 staining were graded as follows: no staining = 0; weak staining = 1; moderate staining = 2; dense staining = 3. Tissue 1 was scored as “0”, 2 and 6 scored as “1”, 3 and 7 scored as “2”, 4 and 8 scored as “3”. Figure S3. Expression of IGFBP3 in a penal of human gastric cancer cell lines. Conventional RT-PCR (A) and Western blotting (B) were used to detect expression levels of IGFBP3 in 8 human gastric cancer cell lines (AGS/MKN28/MKN45/NCI-N87/BGC823/HGC27/MGC802/SGC7901) and human gastric epithelial immortalized GES-1 cells. Figure S4. IGFBP3 inhibits gastric cancer cell migration (scratch test). Cell migration was detected by wound-healing assay in BGC823 and SGC7901 cells. The wound in the area of cells was curved when the cell migrated for 12h and 24 h after transfected with negative control siRNA (Ne-si) or IGFBP3 siRNA (I3-si). Representative images were taken with 40 magnification power from triple experiments. Figure S5. Effects of IGFBP3 on E-cadherin and β-catenin in BGC823 cells. Total, soluble and insoluble E-cadherin and β-catenin in BGC823 cells were detected by Western blotting after transfected with negative contr [file pone.0081423.s001.pdf]

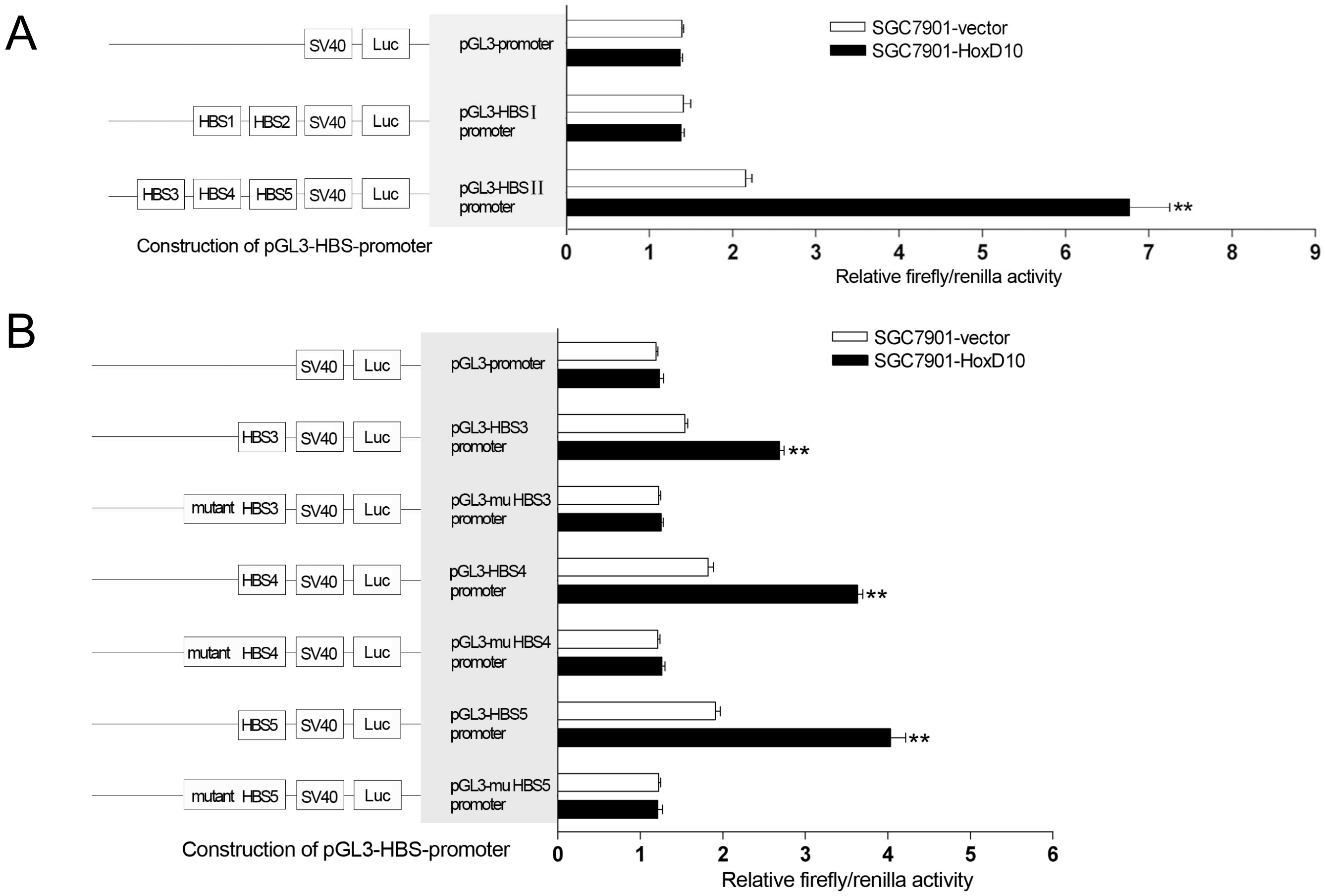

Figure S1. Identification of the regulatory regions of IGFBP3 which are responsible by HoxD10 in SGC7901cells.
(**A**) SGC7901 cells were transfected with pcDNA3.1 empty vector or pcDNA3.1-HoxD10, pGL3-promoter vector or pGL3-HBSI (**II**)-promoter and pRL-TK vector. (**B**) Different oligonucleotides which contain the wild or point mutant sequences of HBS3, HBS4 and HBS5 were cloned into pGL3-HBS-promoter. Relative firefly activity was expressed normalized to renilla activity in pRL-TK vector. All experiments were performed in triplicate. ** indicates of p<0.01.

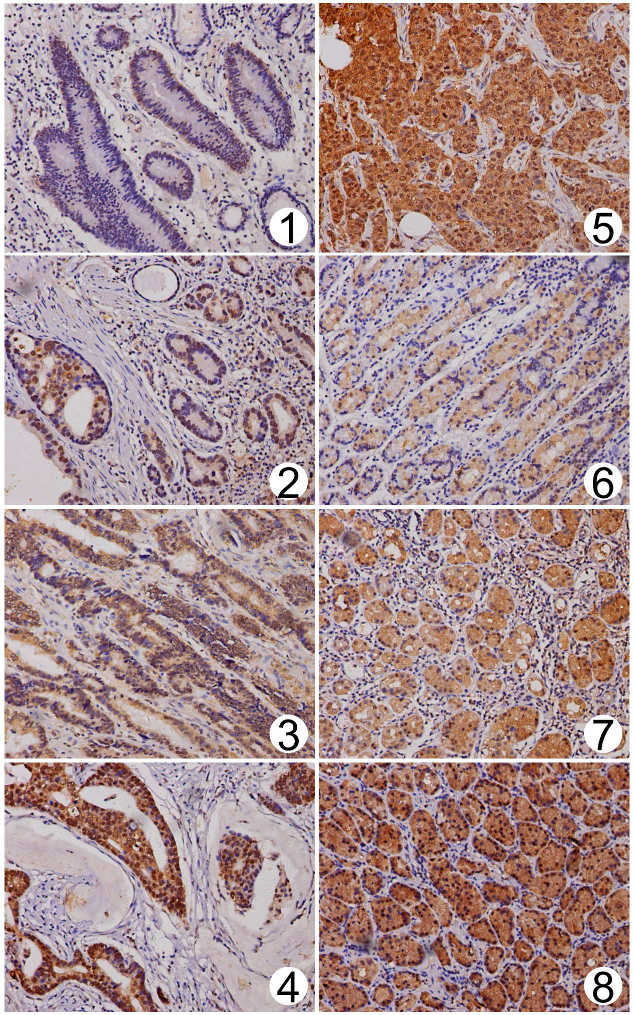

Figure S2. Staining density of IGFBP3 in gastric tissues.

The expression levels of IGFBP3 were detected by immunochemistry analysis in gastric tumor (1-4) and adjacent tumor free tissues (6-8), as well as breast tumor tissue (5, positive control). The intensities of IGFBP3 staining were graded as follows: no staining = 0; weak staining = 1; moderate staining = 2; dense staining = 3. Tissue 1 was scored as “0”, 2 and 6 scored as “1”, 3 and 7 scored as “2”, 4 and 8 scored as “3”.

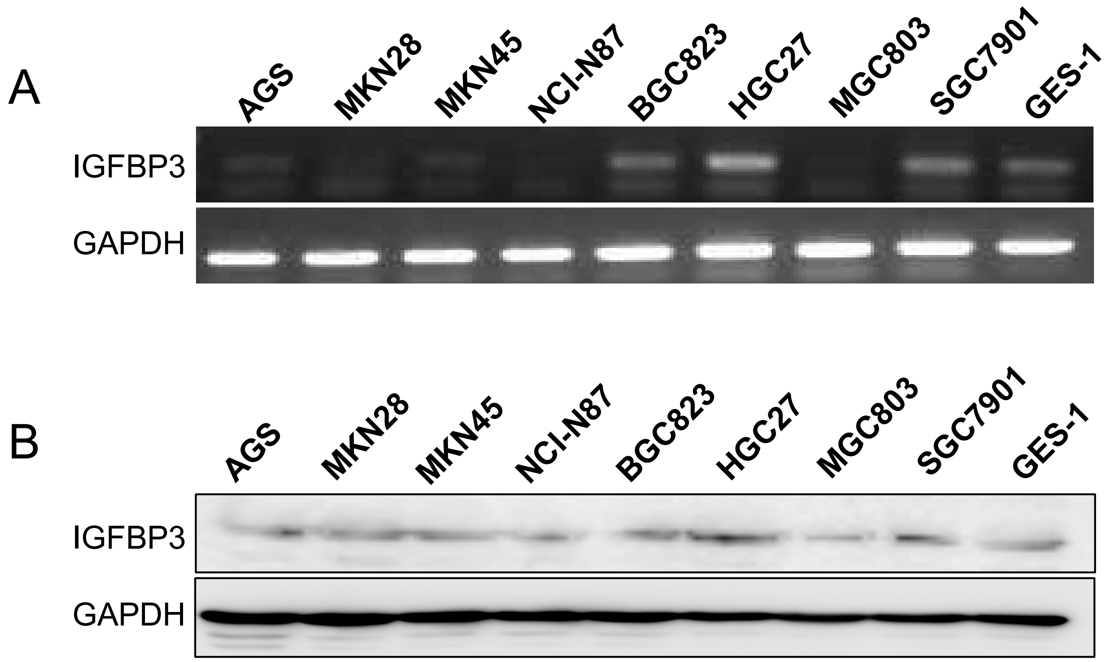

Figure S3. Expression of IGFBP3 in a penal of human gastric cancer cell lines. Conventional RT-PCR (A) and Western blotting (B) were used to detect expression levels of IGFBP3 in 8 human gastric cancer cell lines (AGS/MKN28/MKN45/NCI-N87/BGC823/HGC27/MGC802/SGC7901) and human gastric epithelial immortalized GES-1 cells.

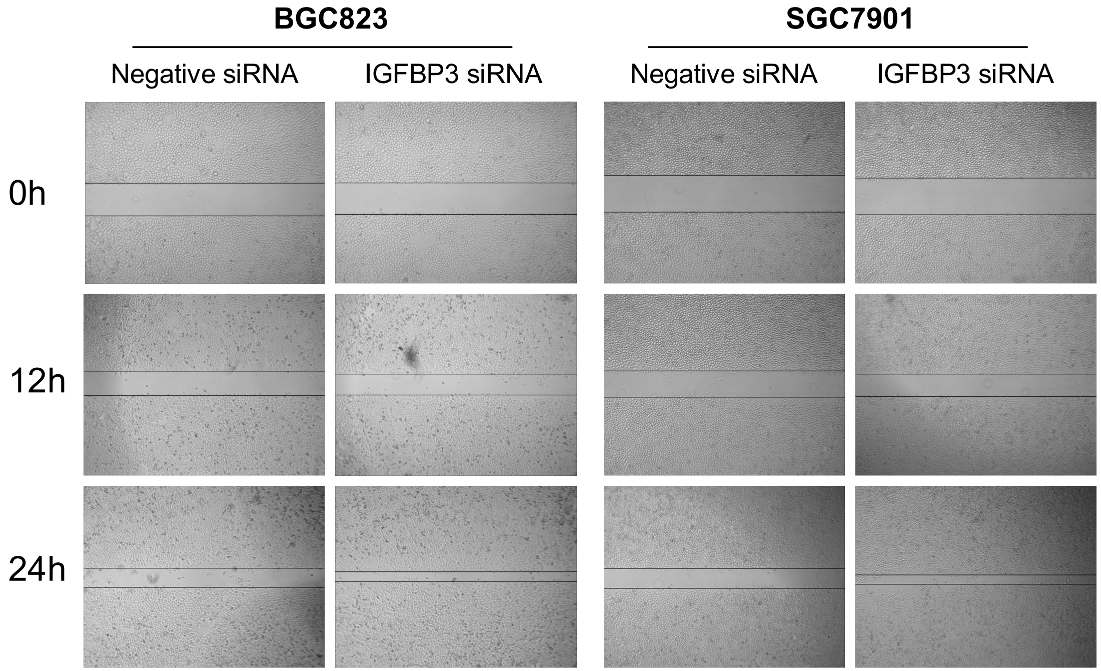

Figure S4. IGFBP3 inhibits gastric cancer cell migration (scratch test).
Cell migration was detected by wound-healing assay in BGC823 and SGC7901 cells. The wound in the area of cells was curved when the cell migrated for 12h and 24 h after transfected with negative control siRNA (Ne-si) or IGFBP3 siRNA (I3-si). Representative images were taken with 40 magnification power from triple experiments.

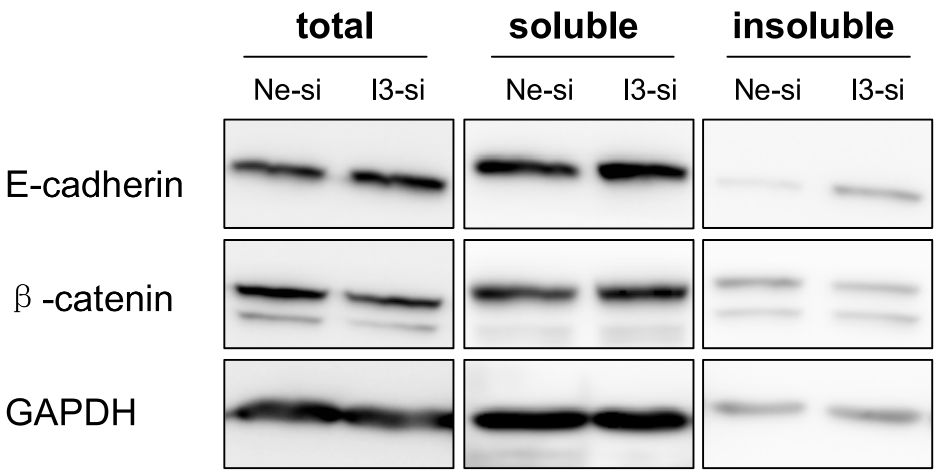

Figure S5. Effects of IGFBP3 on E-cadherin and β -catenin in BGC823 cells.
Total, soluble and insoluble E-cadherin and β -catenin in BGC823 cells were detected by Western blotting after transfected with negative control siRNA (Ne-si) or IGFBP3 siRNA (I3-si).
