# Supplementary material for: IGFBP3, a Transcriptional Target of Homeobox D10, Is Correlated with the Prognosis of Gastric Cancer
Source: PLoS One. 2013 Dec 27;8(12):e81423. doi: 10.1371/journal.pone.0081423 (PMC3873913; doi:10.1371/journal.pone.0081423)
Supplement: Table S1 — Possible HoxD10 binding sites at the promoter region of IGFBP3. (DOC) [file pone.0081423.s002.doc]

**Table S1** Possible HoxD10 binding sites at the promoter region of IGFBP3.

| Names | | Start position | End position | Sequence (5'-3') |
| --- | --- | --- | --- | --- |
| HBSI | HBS1 | -2191 | -2182 | AATAAAACAA |
|  | HBS2 | -2111 | -2102 | AATAAAAAGT |
| HBSII | HBS3 | -1700 | -1691 | TCTTTTTATT |
|  | HBS4 | -1418 | -1409 | ATTTGCTATT |
|  | HBS5 | -953 | -944 | CTTTATTATT |
